# Epigenetics of violence against women: a systematic review of the literature

**DOI:** 10.1093/eep/dvae012

**Published:** 2024-08-10

**Authors:** Paolo Bailo, Andrea Piccinini, Giussy Barbara, Palmina Caruso, Valentina Bollati, Simona Gaudi

**Affiliations:** Section of Legal Medicine, School of Law, University of Camerino, Camerino 62032, Italy; Department of Biomedical Sciences for Health, Università degli Studi di Milano, Milan 20100, Italy; Service for Sexual and Domestic Violence (SVSeD), Fondazione IRCCS Ca’ Granda Ospedale Maggiore Policlinico, Milan 20100, Italy; Service for Sexual and Domestic Violence (SVSeD), Fondazione IRCCS Ca’ Granda Ospedale Maggiore Policlinico, Milan 20100, Italy; Gynecology Emergency Unit, Fondazione IRCCS Ca’ Granda Ospedale Maggiore Policlinico, Milan 20100, Italy; Department of Clinical Sciences and Community Health, Dipartimento di Eccellenza 2023-2027, University of Milan, Milan 20122, Italy; Department of Biomedical Sciences for Health, Università degli Studi di Milano, Milan 20100, Italy; Department of Clinical Sciences and Community Health, Dipartimento di Eccellenza 2023-2027, University of Milan, Milan 20122, Italy; Department of Environment and Health, Italian National Institute of Health, Rome 00161, Italy

**Keywords:** sexual violence, intimate partner violence, epigenetics, PTSD, stress-related disorders

## Abstract

Violence against women is a pervasive global issue with profound impacts on victims’ well-being, extending across cultural boundaries. Besides immediate physical harm, it triggers mental health consequences such as post-traumatic stress disorder (PTSD). Indeed, it is the trauma experienced during a violent event that can lead to epigenetic modifications, ultimately contributing to the onset of PTSD. While research on the epigenetic effects of trauma initially focused on war veterans and disaster survivors, there is a dearth of studies on violence against women. In this article, we performed a systematic review aimed to fill this gap, examining existing studies on the epigenetic impact of violence on women. The review assessed sample sizes, study validity, and gene-specific investigations. Currently, there is insufficient data for a comprehensive meta-analysis, highlighting a nascent stage in understanding this complex issue. Future research is crucial for deeper insights into the epigenetic mechanisms related to violence against women, contributing to improved interventions and support healthcare systems for affected individuals.

## Introduction

Violence against women is a pervasive and alarming health and social issue, with profound repercussions on the well-being of victims. This global phenomenon extends its dark reach across geographical and cultural boundaries, leaving an indelible mark on the fabric of societies. In the context of the European Union, it is disconcerting to acknowledge that a significant proportion of women, estimated to be at least one in three, have experienced some form of violence. Also, the World Health Organization reports that ∼30% of women worldwide have experienced some form of violence, with serious consequences on their physical and psychological health [1]. Shockingly, a recent observational study suggests that this prevalence could be even higher, reaching a staggering 51.7% [2].

The main types of violence against women can be generally classified into sexual violence and intimate partner violence (IPV). Sexual violence is ‘forcing or attempting to force a partner to participate in a sexual act, sexual contact, or nonphysical sexual event (e.g. sexting) when the partner is not or cannot be consenting’. IPV is ‘the abuse or assault that occurs in a romantic relationship’. The term ‘intimate partner’ refers to both current and former spouses and dating partners and can include physical violence, sexual assault, stalking, and economic and psychological violence [3].

Beyond the immediate physical harm inflicted, violence against women has far-reaching consequences [4–6], delving into the realm of mental health. The traumatic nature of these events often propels victims into the throes of post-traumatic stress disorder (PTSD), a debilitating condition that disrupts their psychological well-being [7].

Extensive research highlights how traumatic experiences can impact the epigenetic level, causing changes in genome regulation and gene expression [8, 9]. This process allows the body to respond to trauma by activating defence mechanisms. However, when these mechanisms malfunction, it can lead to the development of PTSD and similar disorders.

The relationship between trauma and epigenetics is a complex mechanism, where genes adapt to environmental stressors. The resulting genomic changes not only influence immediate stress responses but also contribute to long-term effects on mental health [10, 11]. Understanding these molecular processes provides insight into the intricate connection between experiences and the regulation of our genetic material.

Initial investigations into the epigenetic responses to traumatic events primarily centred on war veterans [12] and survivors of natural disasters [13], with subsequent attention given to Holocaust/genocide survivors [14, 15]. These studies initially concentrated on examining DNA methylations and later expanded their scope to include the exploration of microRNAs [16] and histone proteins [17]. Furthermore, numerous studies employing animal models are documented in the literature, demonstrating significant advantages for the investigation of brain cells. Research on gene deregulation following PTSD in these models consistently indicates a potential role for the corticotropin-releasing hormone pathway [18, 19]. However, there remains a notable scarcity of research on epigenetic modifications stemming from experiences of violence against women.

On these premises, we conducted a systematic literature review to examine the existence of relevant studies and assess the current situation on the relation between gender violence against women and epigenetic modifications. Our primary aim was to identify studies that explore the epigenetic impact of traumatic experiences related to violence in adulthood and highlighting the specific genes under scrutiny. Additionally, we examined the sample size and validity of the studies, uncovering critical issues that emerged during our assessment.

## Searching strategy

The research was carried out on the scientific literature between January 2001 and November 2023 in the online databases of PubMed, Scopus, and Web of Science (WoS). The types of study objects of interest were original articles, review articles, book chapters, conference papers, editorial materials, proceeding papers, and meeting abstracts. The PubMed database search was performed by using the following search string: (‘sexual violence’ OR ‘sexual assault’ OR ‘rape’ OR ‘sexual abuse’ OR ‘intimate partner violence’) AND (‘epigenetics’ OR ‘DNA methylation’ OR ‘histone modification’ OR ‘epigenetic regulation’ OR ‘epigenetic changes’ OR ‘Micro RNA’).

We conducted the search on Scopus by entering the following search terms: ‘sexual violence’, ‘sexual assault’, ‘rape’, ‘sexual abuse’, and ‘intimate partner violence’ and combined them with the operator ‘OR’. Then, we performed a new search by inserting the following terms: ‘epigenetics’, ‘DNA methylation’, ‘histone modification’, ‘epigenetic regulation’, ‘epigenetic changes’, and ‘Micro RNA’ and combined them with the operator ‘OR’. We set ‘title-abstract-keywords’ as the tag field. Finally, we combined the results of the two searches with the ‘AND’ operator. The search on WoS was conducted similarly to the search on Scopus, the only difference being that we entered ‘title-abstract’ as the tag field.

Since only published data were considered, the current research project was exempt from institutional review board approval.

We performed a preliminary skimming independently: each author read the abstracts of the articles found and identified those they considered useful for the review. At the end of the preliminary evaluation procedure, the authors discussed the various articles, debating the suitability of the individual papers. At the end of the selection phase, the authors read all the articles in order to collect the data for the review.

## Selection criteria

The research initially provided 681 results. Specifically, 89 articles were found in PubMed, 233 in Scopus, and 359 in WoS. We made an initial preselection by removing duplicate articles (*n* = 232), articles not written in English (*n* = 4), and articles for which the full text was not available (*n* = 3). We then read the abstracts of the remaining 442 articles in order to identify articles suitable for reading the full text. At this stage, we decided to include only those articles that specifically explored epigenetic modifications in adult female victims of violence and removed articles on female victims younger than 18 years. After reading the abstracts, we excluded 391 articles as not relevant to the purposes of the review. Specifically, 215 articles were related to areas completely unrelated to the topic of interest (e.g. epigenetics on rape seed and forensic techniques for epigenetic analysis), 33 articles discussed the epigenetic modification of stress-related psychiatric disorders without explicitly addressing violence, and 190 articles discussed the epigenetic modification in children and adolescent victims of violence.

We then proceeded to read the full text of the remaining four articles that were included in the final review. To conduct this systematic review, we adhered to the Preferred Reporting Items for Systematic reviews and Meta-Analyses guidelines [20].

## Quality evaluation

The Scale for the Assessment of Narrative Review Articles (SANRA) [21] was employed for the quality check of the selected studies. The overall quality was determined as poor (score 0–6), moderate (7–9), and excellent (10–12). The four articles were found to be of excellent quality. The results of the SANRA are reported in Table 1.

**Table 1.** The SANRA score for the quality assessment of selected studies for the review.

Reference and year of publication	Justification of the article’s importance for the readership	Statement of concrete aims or formulation of questions	Description of the literature search	Referencing	Scientific reasoning	Appropriate presentation of data	Total score
Checknita *et al*., 2018 [22]	2	2	1	2	2	2	11
Checknita *et al*., 2022 [23]	2	2	1	2	2	2	11
Nöthling *et al*., 2021 [24]	2	2	2	2	2	2	12
Piccinini *et al*., 2023 [25]	2	2	2	2	2	2	12

**Figure 1.**
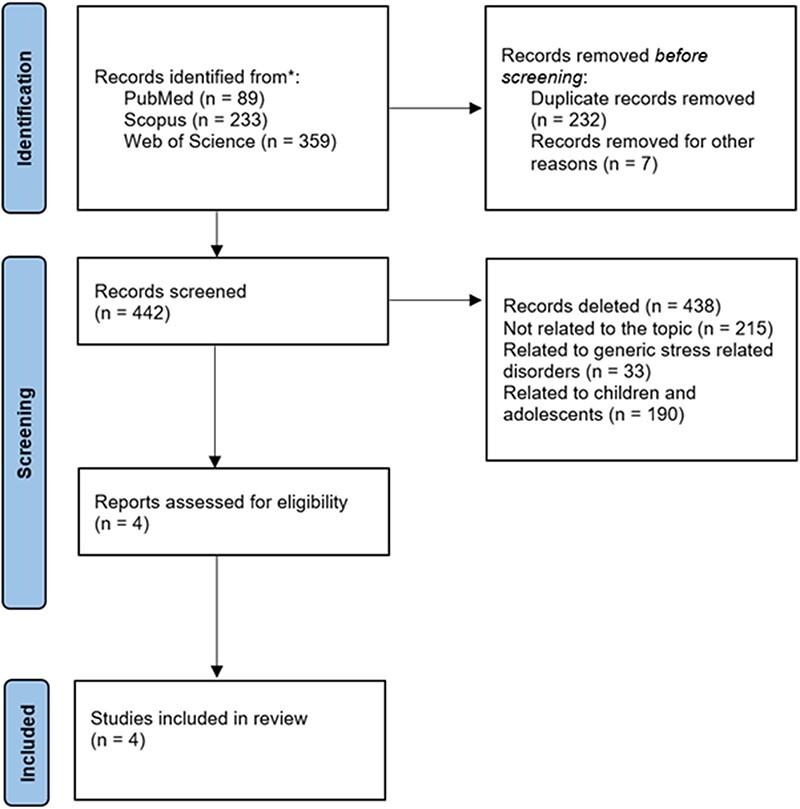
Review search strategy.

Figure 1 illustrates the article selection process.

## Summary of article pool

The search identified four articles suitable for inclusion in the review. All four articles were published between 2018 and 2023. The socio-environmental and cultural background of the articles was Europe and South Africa. Regarding the types of articles, they were original research articles in which the authors conducted an analysis to discover the epigenetic signature of violence against women. Table 2 summarizes the main characteristics of the reviewed articles.

**Table 2.** Summary of the content of the four articles included in the review.

Reference and year of publication	Socio-environmental context	Type of article	Title	Number and types of cases	Overall results
Checknita *et al*., 2018 [22]	Europe	Research Article	Associations of MAOA gene first exon methylation with sexual abuse and current depression in women	Sample containing 114 women with 65 childhood physical abuse/sexual abuse and 26 only sexual abuse (age range median 22 years)	Single-gene methylation level analysis (*MAOA*) on saliva. Sexual abuse was associated with hypermethylation of MAOA exon. Small sample and lacked a distinction between different types of violence. The study does not specify the time elapsed between violent events and analysis.
Checknita *et al*., 2022 [23]	Europe	Research Article	Associations of age, sex, sexual abuse, and genotype with MAOA gene methylation	Sample containing 252 women with 64 cases of sexual abuse (age range 15–62 years)	Single-gene methylation level analysis (*MAOA*) on saliva. Methylation levels were slightly increased in women who experienced sexual abuse. Small sample and lacked a distinction between different types of violence. The study does not specify the time elapsed between violent events and analysis.
Nöthling *et al*., 2021 [24]	South Africa	Research Article	Genome-wide differentially methylated genes associated with posttraumatic stress disorder and longitudinal change in methylation in rape survivors	Sample containing 96 women exposed to sexual violence (age range 18–40 years)	Epigenome-wide analysis with validation of *BRSK2* and *ADCYAP1* and 6 months follow-up on peripheral blood. One differentially methylated CpG site and 34 differentially methylated regions were associated with PTSD status. Decreased *BRSK2 and ADCYAP1* methylation may be associated with more severe PTSD. Small sample with only raped subjects. The study does specify the time elapsed between violent events and analysis.
Piccinini *et al*., 2023 [25]	Europe	Research Article	Violence against women and stress-related disorders: seeking for associated epigenetic signatures, a pilot study	Sample containing 112 women with 62 cases of sexual abuse (age range 18–65 years)	Ten-gene methylation level analysis (*ADCYAP1, BDNF, CRHR1, DRD2, FKBP5, IGF2, LSD1/KDM1A, NR3C1, PRTFDC1, SLC6A4*) on peripheral blood. Violence against women was associated with hypermethylation of *BDNF*, *DRD2*, and *IGF2.* Pilot study and small sample. A distinction between types of violence is present. The study does specify the time elapsed between violent events and analysis.

## Statement on the utilization of generative AI software

No data or contents in this article were generated by AI. All information was manually written and discussed by the authors. To enhance fluency, syntax, and grammar, DeepL, ChatGPT 3.5, and Grammarly (free version) were used exclusively for text editing. Additionally, the meaning of each sentence was double-checked by all authors. In cases of discrepancies, the original version was restored.

## Results

Existing research highlights a significant gap in the investigation of epigenetic modifications specifically related to violence against women and the subsequent psychological disorders. The available literature predominantly concentrates on potential epigenetic alterations in child/adolescent victims of violence, neglecting the adult population. Out of the articles addressing the issue, only a limited number, precisely four, deal specifically with epigenetic changes in the adult population. However, these studies employ various analytical approaches, and their focus is limited to the methylation aspect of the epigenetic mechanism.

Checknita’s research group conducted two studies [22, 23], both focused on the investigation of the methylation patterns of the gene responsible for MAOA, together with instances of violence against women.

MAOA catalyses the oxidative deamination of amines, such as dopamine, norepinephrine, and serotonin. This enzyme regulates normal synaptic function in the brain and is also expressed in cardiomyocytes as part of the stress response. Alterations in MAOA expression are associated with behavioural and neurological disorders [26–30].

The initial study, carried out in 2018, involved a sample of 114 women, 65 of whom had experienced sexual assault and/or physical violence during childhood. Additionally, there were 26 cases of sexual assault only. The average age of participants was 22 years, and DNA was extracted from the saliva samples. Interestingly, it was found that in women who had been raped, the MAOA gene exhibited hypermethylation in the first exon. The limitations of this study are the small sample size and that women experienced physical violence during their minor age.

The second study, published in 2021, followed similar methods to the first one. It involved a larger sample of 252 women, 64 of whom had reported sexual abuse, spanning ages 15–62 years. Once again, DNA was extracted from the saliva samples. The results of this study mirrored those of the previous one, indicating a slight increase in methylation levels in both exons and introns of the MAOA gene in women who had experienced sexual violence. However, the low number of participants remained a significant limitation. Furthermore, both studies undertaken by Checknita’s research group fall short in providing a clear differentiation between distinct types of violence, such as sexual assault and IPV. Additionally, there is no specification regarding the time elapsed between the occurrence of the violent events and the subsequent analysis.

The 2021 study by Nöthling *et al*. [24] examines the epigenetic mechanism of methylations but employs a distinct analytical approach. Specifically, it investigates alterations in methylation patterns among victims of violence, analysing the entire genome 3 months after the traumatic event. Following gene verification, the study validates the *brain-specific serine/threonine-protein kinase 2 (BRSK2)* and *ADCYAP1* genes, showcasing the methylation trends of these genes over the subsequent 6 months.

*BRSK2* facilitates various functions, such as Adenosine Triphosphate (ATP) binding, ATPase binding, and protein kinase activity. It is crucial for neuron polarization and axonogenesis, cell cycle progression, and insulin secretion [31]. This enzyme is highly expressed in the hippocampus and is associated with PTSD and memory formation [32]. Changes in *BRSK2* methylation and expression can impact the levels of PTSD-related neurotransmitters [33]. It is also expressed in pancreatic cells, and its deregulation could be related to abnormal blood glucose levels with increased risk of diabetes and cardiovascular disorders in PTSD [34].

The protein product of *ADCYAP1* is pituitary adenylate cyclase-activating polypeptide (PACAP), which acts as a key regulator of the Hypothalamic–pituitary–adrenal axis and the stress response. PACAP stimulates the release of corticotropin-releasing hormone in the hypothalamus and promotes the release of catecholamines in the adrenal medulla and catecholamine-synthesizing enzymes (Phenylethanolamine N-methyltransferase and Tyrosine hydroxylase) [35]. Elevated PACAP levels in the blood are linked to the most severe PTSD symptoms and appear to increase activity in the amygdala and hippocampus in individuals with PTSD [36].

The study enrolled a cohort of 96 subjects who experienced sexual violence in the 20 days preceding the baseline visit, aged between 18 and 40 years, with efforts made to maintain a homogeneous ethnical sample. Additionally, the study aimed to include subjects both diagnosed with PTSD and those without, enhancing the exploration of differences at the epigenetic level. DNA analysis was conducted on peripheral blood. The results, obtained through a comprehensive genome-wide approach, identified one differentially methylated CpG site and 34 differentially methylated regions associated with PTSD status 3 months post incident. Subsequent analyses revealed a decrease in *BRSK2* methylation levels in the presence of PTSD, with a more pronounced decrease correlating with greater symptom severity. Although this result was validated, replication was unsuccessful. Conversely, the reduction in *ADCYAP1* methylation levels was observed in the presence of PTSD but remained unvalidated and unreplicated. The study acknowledges limitations, particularly the small participant size affecting statistical significance. Additionally, the importance of replicating the results in subsequent studies is emphasized to ensure the robustness of the observed trends.

The study conducted by Piccinini *et al*. [22], published in 2023, investigated the methylation patterns of 10 candidate genes (*ADCYAP1, BDNF, CRHR1, DRD2, FKBP5, IGF2, LSD1/KDM1A, NR3C1, PRTFDC1, and SLC6A4*) associated with stress-related disorders, as PTSD, in women exposed to IPV and sexual violence. The research involved 112 women aged 18–65 years, including 62 subjected to violence (13 to sexual violence and 49 to IPV). DNA was extracted from blood samples collected at the antiviolence centre, with clear knowledge of the time gap between the violent event and collection. The initial design involved a follow-up for monitoring the epigenetic changes in the following months, but high participant dropout led to its abandonment. The pilot study suggests hypermethylation in *BDNF*, *DRD2*, and *IGF2* in female violence victims (both sexual and IPV).

The *BDNF* gene, which encodes brain-derived neurotrophic factor, is a key growth factor and regulator of synaptic transmission and neuroplasticity. It plays a role in stress response, learning, and memory [37]. Increased methylation of this gene is stress related and has been observed in individuals with PTSD [38].

The *DRD2* gene encodes the dopamine receptor D2 protein. Studies have shown that sexual violence can increase the methylation of *DRD2*, potentially reducing dopaminergic activity at D2 receptors. This suggests a persistent epigenetic modification at this gene locus following sexual abuse [39].

*IGF2* is a gene regulated by genomic imprinting that encodes insulin-like growth factor 2, a key regulator of fetal and placental growth. Additionally, *IGF2* influences various brain functions including memory, depression, and autism [40] and impacts the expression of *BDNF* [41]. Changes in *IGF2* expression have been observed in individuals with PTSD [42]. However, the small sample size limits the statistical significance of the results.

## Discussion

Research on the epigenetic mechanisms of violence against women is still an emerging field, marked by a scarcity of comprehensive studies. However, the significance of this work lies not only in analysing current findings but also primarily in raising awareness about the necessity of understanding the epigenetic impacts of violence. In this context, our inquiry aims to lay the groundwork for future investigations as we recognize the crucial importance of delving into these mechanisms to enhance therapies and promote a more empathetic and personalized care perspective for survivors.

Addressing violence against women necessitates a comprehensive approach that goes beyond immediate intervention. It involves not only treating the visible wounds but also providing long-term support for the psychological and physical recovery of survivors. Recognizing the medical dimensions of this issue is imperative for healthcare professionals, policymakers, and society as a whole to work collaboratively towards eradicating this pervasive problem and promoting the full holistic well-being of women.

The widespread issue of violence against women is progressively recognized as a significant medical concern, given its profound and enduring impact on women’s health across the short-, medium-, and long-term durations. This multifaceted problem not only inflicts immediate physical injuries but also engenders a cascade of adverse health outcomes that persist over time. Violence against women has immediate consequences causing physical trauma such as injuries, fractures, and contusions. Beyond visible injuries, there are hidden psychological scars leading to distress, anxiety, and depression. In the medium term, it affects reproductive health, mental well-being, and overall quality of life. Women who have experienced violence may struggle in establishing and maintaining healthy relationships, perpetuating emotional and psychological distress [43].

Furthermore, the long-term effects are alarming as chronic exposure to violence is linked to various health conditions, including cardiovascular diseases, autoimmune disorders, and chronic pain syndromes [4–6]. Survivors also face an increased risk of engaging in harmful behaviours, such as substance abuse and self-harm. Moreover, a particularly harmful and widespread form of violence is still underexplored from an epigenetic perspective, i.e. the use of rape as a weapon of war. In this case, the traumatic consequences on the victims are of huge impact on their psychological and physical health, although very little is explored [44].

Recognizing the existing constraint within health policies concerning the management of events related to violence against women in the medium to long term, our curiosity investigation led us to explore the realm of personalized precision medicine. We sought to investigate whether the current scientific literature has established a robust foundation in approaching the phenomenon of violence against women from the perspective of epigenetics.

Existing literature has already highlighted studies demonstrating specific epigenetic changes induced by major traumas, such as those observed in war veterans or survivors of natural disasters. The scientific literature under examination predominantly focuses on the epigenetics of trauma in child victims of violence. However, studies addressing this issue in adults are notably limited, with only four identified in our review. These studies, while all focused on the common epigenetic mechanism of methylations, exhibit significant disparities in their methodologies. The studies examined display a diverse range of investigative approaches, ranging from the analysis of individual gene methylations to the study of the entire epigenome. It is important to note that only one of these studies attempts to distinguish between sexual violence and IPV. However, its small sample size limits its statistical power to detect significant differences in the methylation patterns of victims. The other studies focus solely on sexual violence and do not investigate IPV.

The low number of participants in these studies highlights the challenges in recruiting women who are victims of violence. These women are often traumatized and vulnerable, both psychologically and physically. When a victim seeks help at an antiviolence centre, the staff must prioritize immediate diagnostic and therapeutic procedures to provide the best support possible. Under these circumstances, explaining a study and obtaining consent to collect samples is quite difficult. Additionally, conducting follow-up studies on epigenetic patterns is challenging due to a high dropout rate as it is hard to convince these vulnerable individuals to return frequently to the centre.

Another aspect to consider is the high prevalence of drug and alcohol use, as well as other psychiatric disorders, among these vulnerable individuals [5, 45–47]. While it is possible, it is unlikely to find a healthy subject experiencing rape for the first time who then exhibits epigenetic modifications solely due to the violent event. Therefore, when analysing results, it is crucial to account for these additional factors that could distort the findings. A comprehensive collection of medical and psychological history is essential for accurate analysis.

Considering the limited attention given to delayed repercussions in the context of violence against women, a transformative medical paradigm centred on the individual is starting to emerge. This innovative approach seeks to tailor healthcare interventions to meet each individualized need. At its core, this paradigm draws its strength from the examination of the epigenetic signature written in the DNA by violence. The shift towards this personalized healthcare paradigm represents a profound departure from the ‘one-size-fits-all’ approach that has, regrettably, been the norm for addressing violence against women. Rather than treating survivors as a homogenous group, it acknowledges the profound diversity among them, considering not only the physical and psychological traumas they have suffered but also the interplay with their DNA that influences their health outcomes. Epigenetics, in particular, offers a fascinating perspective as it encompasses the study of modifications to gene expression that occur without alterations to the underlying DNA sequence. These epigenetic changes have the potential to provide crucial insights into how the trauma of violence against women can leave indelible marks on their physical and mental well-being, understanding how they may contribute to the lasting health disparities experienced by survivors.

By the unique epigenetic signatures of individual survivors, healthcare professionals can begin to explore and counteract the underlying mechanisms through which violence against women exerts its long-lasting effects, developing personalized interventions that consider and tailor treatments to address each woman’s specific needs. This approach not only holds the promise of more effective healthcare interventions but also embodies a profound shift towards a more empathetic model of care.

## Conclusion

The exploration of the repercussions of violence against women through an epigenetic lens is in its early stages. Currently, there are insufficient studies to facilitate a comprehensive meta-analysis. We envisage that future research endeavours will shed more light on this critical and sensitive subject, enhancing our understanding of the phenomenon over time, thus enabling more appropriate therapies in a multidisciplinary and personalized perspective.

Violence against women is a highly sensitive and urgent issue with significant social repercussions. Personalized medicine offers a valuable approach to addressing and managing each individual case effectively. However, conducting studies in this area is challenging due to limited resources and difficulties in obtaining samples. Thus, it is crucial to undertake extensive collaborative research efforts to advance our understanding and improve interventions.

## Data Availability

There are no new data associated with this article.

## References

[1] World Health Organization . Global and Regional Estimates of Violence Against Women: Prevalence and Health Effects of Intimate Partner Violence and Non-Partner Sexual Violence. World Health Organization Publications. Geneva, Switzerland. https://www.who.int/publications/i/item/9789241564625 (20 March 2024, date last accessed).

[2] Barbier A, Chariot P, Lefèvre T. Intimate partner violence against ever-partnered women in Europe: prevalence and associated factors-results from the violence against women EU-wide survey. *Front Public Health* 2022;10:1033465. 10.3389/fpubh.2022.1033465 PMC9755339 36530735

[3] U.S. Centers for Disease Control and Prevention (CDC) . About Intimate Partner Violence. https://www.cdc.gov/intimate-partner-violence/about/index.html (16 July 2024, date last accessed).

[4] Chandan JS, Thomas T, Bradbury-Jones C et al. Risk of cardiometabolic disease and all-cause mortality in female survivors of domestic abuse. *JAMA* 2020;9:e014580. 10.1161/JAHA.119.014580 PMC7070197 32063124

[5] Stubbs A, Szoeke C. The effect of intimate partner violence on the physical health and health-related behaviors of women: a systematic review of the literature. *Trauma Violence Abuse* 2022;23:1157–72. 33541243 10.1177/1524838020985541

[6] Reingle Gonzalez JM, Jetelina KK, Olague S et al. Violence against women increases cancer diagnoses: results from a meta-analytic review. *Prev Med* 2018;114:168–79. 29981792 10.1016/j.ypmed.2018.07.008

[7] Stewart DE, Vigod SN. Update on mental health aspects of intimate partner violence. *Med Clin North Am* 2019;103:735–49. 31078204 10.1016/j.mcna.2019.02.010

[8] Conching AKS, Thayer Z. Biological pathways for historical trauma to affect health: a conceptual model focusing on epigenetic modifications. *Soc Sci Med* 2019;230:74–82. 30986608 10.1016/j.socscimed.2019.04.001

[9] Walsh K, Galea S, Koenen KC. Mechanisms underlying sexual violence exposure and psychosocial sequelae: a theoretical and empirical review. *Clin Psychol* 2012;19:260–75. 10.1111/cpsp.12004 PMC4353631 25762853

[10] Mathews HL, Janusek LW. Epigenetics and psychoneuroimmunology: mechanisms and models. *Brain Behav Immun* 2011;25:25–39. 20832468 10.1016/j.bbi.2010.08.009 PMC2991515

[11] Argentieri MA, Nagarajan S, Seddighzadeh B et al. Epigenetic pathways in human disease: the impact of DNA methylation on stress-related pathogenesis and current challenges in biomarker development. *eBioMedicine* 2017;18:327–50. 28434943 10.1016/j.ebiom.2017.03.044 PMC5405197

[12] Verhoeven JE, Yang R, Wolkowitz OM et al. Epigenetic age in male combat-exposed war veterans: associations with posttraumatic stress disorder status. *Mol Neuropsychiatry* 2018;4:90–9. 30397597 10.1159/000491431 PMC6206951

[13] Hong C, Efferth T. Systematic review on post-traumatic stress disorder among survivors of the Wenchuan earthquake. *Trauma Violence Abuse* 2016;17:542–61. 26028651 10.1177/1524838015585313

[14] Yehuda R, Daskalakis NP, Bierer LM et al. Holocaust exposure induced intergenerational effects on FKBP5 methylation. *Biol Psychiatry* 2016;80:372–80. 26410355 10.1016/j.biopsych.2015.08.005

[15] Vukojevic V, Coynel D, Ghaffari NR et al. NTRK2 methylation is related to reduced PTSD risk in two African cohorts of trauma survivors. *Proc Natl Acad Sci U.S.A*. 2020;117:21667–72. 32817534 10.1073/pnas.2008415117 PMC7474665

[16] Snijders C, de Nijs L, Baker DG et al. MicroRNAs in post-traumatic stress disorder. *Curr Top Behav Neurosci* 2018;38:23–46. 29063484 10.1007/7854_2017_32

[17] Bonomi RE, Girgenti M, Krystal JH et al. A role for histone deacetylases in the biology and treatment of post-traumatic stress disorder: what do we know and where do we go from here? *Complex Psychiatry* 2022;8:13–27. 36545044 10.1159/000524079 PMC9669946

[18] Ell MA, Schiele MA, Iovino N et al. Epigenetics of fear, anxiety and stress – focus on histone modifications. *Curr Neuropharmacol* 2024;22:843–65. 36946487 10.2174/1570159X21666230322154158 PMC10845084

[19] Núñez-Rios DL, Martínez-Magaña JJ, Nagamatsu ST et al. Cross-species convergence of brain transcriptomic and epigenomic findings in posttraumatic stress disorder: a systematic review. *Complex Psychiatry* 2023;9:100–18. 37404872 10.1159/000529536 PMC10315001

[20] Moher D, Shamseer L, Clarke M et al. Preferred reporting items for systematic review and meta-analysis protocols (PRISMA-P) 2015 statement. *Syst Rev* 2015;4:1. 10.1186/2046-4053-4-1 PMC4320440 25554246

[21] Baethge C, Goldbeck-Wood S, Mertens S. SANRA—a scale for the quality assessment of narrative review articles. *Res Integr Peer Rev* 2019;4:5. 10.1186/s41073-019-0064-8 PMC6434870 30962953

[22] Checknita D, Ekström TJ, Comasco E et al. Associations of monoamine oxidase A gene first exon methylation with sexual abuse and current depression in women. *J Neural Transm* 2018;125:1053–64. 29600412 10.1007/s00702-018-1875-3 PMC5999185

[23] Checknita D, Tiihonen J, Hodgins S et al. Associations of age, sex, sexual abuse, and genotype with monoamine oxidase a gene methylation. *J Neural Transm* 2021;128:1721–39. 34424394 10.1007/s00702-021-02403-2 PMC8536631

[24] Nöthling J, Abrahams N, Toikumo S et al. Genome-wide differentially methylated genes associated with posttraumatic stress disorder and longitudinal change in methylation in rape survivors. *Transl Psychiatry* 2021;11:594. 10.1038/s41398-021-01608-z PMC8604994 34799556

[25] Piccinini A, Bailo P, Barbara G et al. Violence against women and stress-related disorders: seeking for associated epigenetic signatures, a pilot study. *Healthcare* 2023;11:173. 10.3390/healthcare11020173 PMC9858929 36673541

[26] Hill J, Nathan R. Childhood antecedents of serious violence in adult male offenders. *Aggress Behav* 2008;34:329–38. 18265408 10.1002/ab.20237

[27] Goldstein RB, Chou SP, Saha TD et al. The epidemiology of antisocial behavioral syndromes in adulthood: results from the national epidemiologic survey on alcohol and related conditions-III. *J Clin Psychiatry* 2017;78:90–8. 27035627 10.4088/JCP.15m10358 PMC5025322

[28] Melas PA, Wei Y, Wong CCY et al. Genetic and epigenetic associations of MAOA and NR3C1 with depression and childhood adversities. *Int J Neuropsychopharmacol* 2013;16:1513–28. 23449091 10.1017/S1461145713000102

[29] McCrory E, De Brito SA, Viding E. The link between child abuse and psychopathology: a review of neurobiological and genetic research. *J R Soc Med* 2012;105:151–6. 22532655 10.1258/jrsm.2011.110222 PMC3343716

[30] Melas PA, Forsell Y. Hypomethylation of MAOA’s first exon region in depression: a replication study. *Psychiatry Res* 2015;226:2–4. 10.1016/j.psychres.2015.01.003 25623016

[31] GeneCards The Human Gene Database . BRSK2 Gene - BR Serine/Threonine Kinase 2. https://www.genecards.org/cgi-bin/carddisp.pl?gene=BRSK2 (16 July 2024, date last accessed).

[32] Ross DA, Arbuckle MR, Travis MJ et al. An integrated neuroscience perspective on formulation and treatment planning for posttraumatic stress disorder: an educational review. *JAMA Psychiatry* 2017;74:407–15. 28273291 10.1001/jamapsychiatry.2016.3325 PMC5504531

[33] Krystal J, Neumeister A. Noradrenergic and serotonergic mechanisms in the neurobiology of posttraumatic stress disorder and resilience. *Brain Res* 2009;1293:13–23. 19332037 10.1016/j.brainres.2009.03.044 PMC2761677

[34] Ryder AL, Azcarate PM, Cohen BE. PTSD and physical health. *Curr Psychiatry Rep* 2018;20:116. 10.1007/s11920-018-0977-9 30367276

[35] Mustafa T . Pituitary adenylate cyclase-activating polypeptide (PACAP). *Adv Pharmacol* 2013;68:445–57. 24054157 10.1016/B978-0-12-411512-5.00021-X

[36] Ressler KJ, Mercer KB, Bradley B et al. Post-traumatic stress disorder is associated with PACAP and PAC1 receptor. *Nature* 2011;470:492–7. 21350482 10.1038/nature09856 PMC3046811

[37] Gray JD, Milner TA, McEwen BS. Dynamic plasticity: the role of glucocorticoids, brain-derived neurotrophic factor and other trophic factors. *Neuroscience* 2012;239:214–27. 22922121 10.1016/j.neuroscience.2012.08.034 PMC3743657

[38] Kim TY, Kim SJ, Chung HG et al. Epigenetic alterations of the BDNF gene in combat-related post-traumatic stress disorder. *Acta Psychiatr Scand* 2017;135:170–9. 27886370 10.1111/acps.12675

[39] Groleau P, Joober R, Israel M et al. Methylation of the dopamine D2 receptor (DRD2) gene promoter in women with a bulimia-spectrum disorder: associations with borderline personality disorder and exposure to childhood abuse. *J Psychiatr Res* 2014;48:121–7. 24157248 10.1016/j.jpsychires.2013.10.003

[40] Tabano S, Colapietro P, Cetin I et al. Epigenetic modulation of the IGF2/H19 imprinted domain in human embryonic and extra-embryonic compartments and its possible role in fetal growth restriction. *Epigenetics* 2010;5:313–24. 20418667 10.4161/epi.5.4.11637

[41] Mellott TJ, Pender SM, Burke RM et al. IGF2 ameliorates amyloidosis, increases cholinergic marker expression and raises BMP9 and neurotrophin levels in the hippocampus of the APPswePS1dE9 Alzheimer’s disease model mice. *PLoS One* 2014;9:e94287. 10.1371/journal.pone.0094287 PMC3986048 24732467

[42] Rusiecki JA, Byrne C, Galdzicki Z et al. PTSD and DNA methylation in select immune function gene promoter regions: a repeated measures case-control study of U.S. Military Service Members. *Front Psychiatry* 2013;4:56. 10.3389/fpsyt.2013.00056 PMC3690381 23805108

[43] Barbara G, Buggio L, Micci L et al. Sexual violence in adult women and adolescents. *Minerva Obstet Gynecol* 2022;74:261–9. 35147019 10.23736/S2724-606X.22.05071-0

[44] Mukwege Foundation . The Red Line Initative. Guidebook on State Obligations for Conflict-Related Sexual Violence. https://www.endcrsv.org/guidebook/introduction/ (19 September 2024, date last accessed).

[45] Bunce A, Blom N, Capelas Barbosa E. Determinants of referral outcomes for victim-survivors accessing specialist sexual violence and abuse support services. *J Child Sex Abus* 2024;33:355–78. 38613828 10.1080/10538712.2024.2341183

[46] White SJ, Sin J, Sweeney A et al. Global prevalence and mental health outcomes of intimate partner violence among women: a systematic review and meta-analysis. *Trauma Violence Abuse* 2024;25:494–511. 36825800 10.1177/15248380231155529 PMC10666489

[47] Honda T, Wynter K, Yokota J et al. Sexual violence as a key contributor to poor mental health among Japanese women subjected to intimate partner violence. *J Womens Health* 2018;27:716–23. 10.1089/jwh.2016.6276 28880713

